# Case report: An unusual presentation of puerperal sepsis

**DOI:** 10.3389/fmed.2022.990731

**Published:** 2022-08-15

**Authors:** Doron Kabiri, Diana Prus, Roie Alter, Gali Gordon, Shay Porat, Yossef Ezra

**Affiliations:** ^1^Department of Obstetrics and Gynecology, Hadassah Medical Center, Faculty of Medicine, Hebrew University of Jerusalem, Jerusalem, Israel; ^2^Department of Pathology, Hadassah Medical Center, Faculty of Medicine, Hebrew University of Jerusalem, Jerusalem, Israel

**Keywords:** maternal sepsis, puerperal sepsis, maternal mortality, infection in pregnancy, Group A Streptococcus

## Abstract

Group A Streptococcus (GAS; *Streptococcus pyogenes*) is a facultative gram-positive coccus, uncommonly colonizing parturient genitalia, where its presence can potentially lead to a life-threatening invasive infection after delivery. GAS infection typically occurs within the first 4 days post-partum and is characterized by high fever, chills, flashing, abdominal pain, and uterine tenderness. Nonetheless, patients with GAS puerperal sepsis may have an unusual presentation, when fever is absent, and the symptoms and signs can be mild, non-specific, and not indicative of the severity of infection. This unusual presentation may lead to a delayed diagnosis and increase the risk for severe puerperal sepsis. Therefore, in these cases, a high index of suspicion and prompt early antibiotic and surgical treatment is crucial to saving the parturient’s life.

## Case notes

**FIGURE 1:**
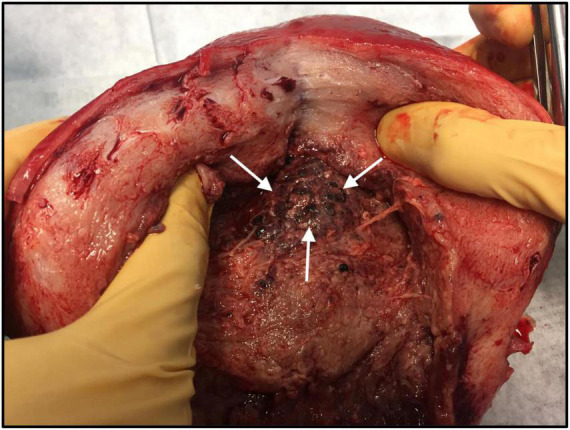
Macroscopic examination of the uterus: Hypertrophic post-partum uterus with necrotic tissue (white arrows), following severe *Streptococcus pyogenes* puerperal sepsis.

**FIGURE 2:**
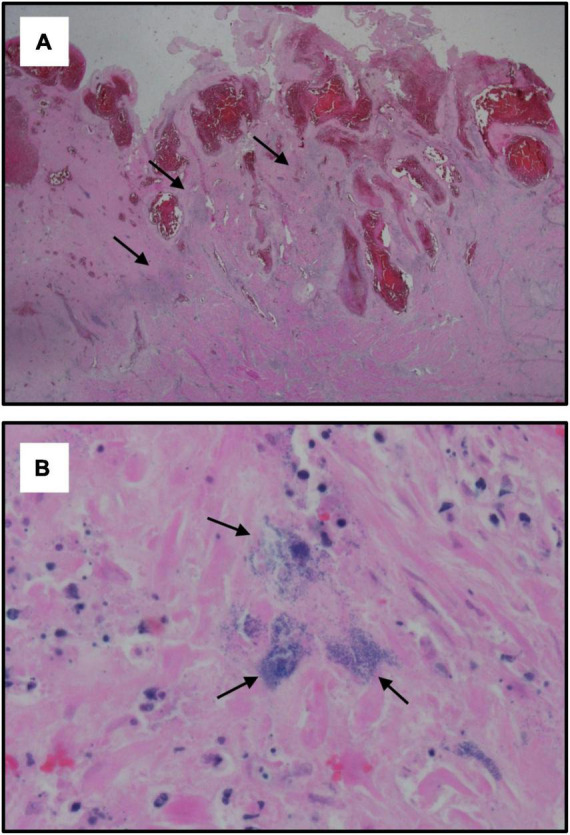
Histological specimens of the uterus. Microscopic examination of the uterus showing: **(A)** Hypertrophic post-partum uterus with acute inflammatory process characterized by extensive areas of recent hemorrhage and dilated congested vessels of the placental site. Black arrows indicate the focus of necrotic myometrium with bacterial colonies (hematoxylin and eosin staining, X1.25); **(B)** Acute inflammation with necrotic myometrium. Black arrows indicate bacterial colonies (hematoxylin and eosin staining, X60).

A 38-year-old woman, gravida 6 para 5, with an unremarkable past medical history presented to labor and delivery in active labor at 39 weeks of gestation and delivered vaginally shortly thereafter. Delivery was uneventful, without regional anesthesia and without perineal tears nor other complications. Twenty-four hours after delivery, the patient developed isolated left lower quadrant pain. Physical examination, abdominal ultrasound, and laboratory tests including complete blood count and basic metabolic panel were unremarkable, and the pain subsided after a bowel movement. On the following day, abdominal pain worsened, while the patient remained afebrile and was hemodynamically stable. Clinical assessment and physical examination of the pelvis and abdomen by the gynecological and surgical teams were unremarkable and revealed no acute distress; the abdomen was soft and non-tender on palpation, and bowel sounds were normal in all four quadrants. Notably, there was a significant discrepancy between the symptoms (referred abdominal pain) and the objective clinical findings. An abdominal and pelvic CT scan demonstrated normal post-partum uterus, endometrium and pelvic organs without signs of acute pathology. A large fecal burden throughout the colon was seen, suggesting possible constipation. Subsequently, 60 h after birth, her clinical condition deteriorated as the patient developed tachycardia with 130 beats per minute, tachypnea with 20 breaths per minute, and blood pressure of 103/65 mmHg. Laboratory values included a white blood cell count of 1.5 × 10^9^/L and C-Reactive Protein (CRP) of 27.1 mg/dl and Lactic acid of 4.05 mmol/L. Creatinine, liver-function tests, and electrolytes were within the normal range. Due to a high clinical suspicion of puerperal sepsis at this point, a wide-spectrum antibiotic regime of ampicillin, clindamycin and gentamicin was initiated, and the patient was transferred to the intensive care unit (ICU). Shortly afterward, the patient became hemodynamically and respiratorily unstable and required sedation, mechanical ventilation, and the use of inotropes to maintain adequate blood pressure. Laboratory results revealed worsening leukopenia, thrombocytopenia, and lactic acidosis. A post-contrast computed tomography scan showed an enlarged uterus with abundant periovarian and peritoneal fluid. Since the presence of pus in the abdomen was suspected and due to the severe clinical deterioration, an emergency exploratory laparotomy was executed, during which 600 ml of thick yellowish-white abdominal fluid was aspirated. The uterus and both ovaries were swollen, necrotic, and covered with fibrin, therefore a total abdominal hysterectomy and bilateral salpingo-oophorectomy were performed. Ovarian preservation was not possible because of severe necrosis. Gross findings of the post-operative pathological specimen showed an ischemic and partially necrotic uterus (Figure 1), while microscopic examination of the uterus revealed a severe acute inflammatory process with necrotic myometrium and bacterial colonies (Figures 2A,B), confirmed later to be *Streptococcus pyogenes* on blood-agar medium culture. Post-operatively, the patient underwent a prolonged recovery period and was discharged without any further obstetrical or gynecological complications.

## Comment

Maternal sepsis or puerperal sepsis is the third leading cause of maternal death in the United States and worldwide (1–4). Maternal sepsis is a life-threatening condition defined as organ dysfunction resulting from infection during pregnancy, childbirth, post-abortion, or post-partum period (5). *Streptococcus pyogenes*, *Escherichia coli*, *Staphylococcus aureus*, Group B Streptococcus, *Streptococcus pneumoniae*, Methicillin-resistant *Staphylococcus aureus* (MRSA), *Clostridium septicum*, and *Morganella morganii* are the predominant pathogens in maternal sepsis. The incidence of maternal sepsis ranges between 0.0001 and 0.004% and maternal mortality due to puerperal sepsis occurs in 9–14% (6–8). It is assumed that about 40% of maternal sepsis cases are preventable if early recognition, early escalation of care, and appropriate antibiotic treatment are applied (9, 10). Risk factors for maternal sepsis include advanced maternal age, preterm premature rupture of the membranes (PPROM) and preterm delivery, multiple gestation pregnancies, cesarean delivery, retained products of conception, post-partum hemorrhage, and maternal comorbidities although maternal sepsis often occurred in patients without risk factors (6).

Group A Streptococcus (GAS; *S. pyogenes*) is a facultative gram-positive coccus, uncommonly colonizing parturient genitalia, where its presence can potentially lead to a life-threatening invasive infection after delivery (11–14). GAS infection typically occurs within the first 4 days post-partum and is characterized by high fever, chills, flushing, abdominal pain, and uterine tenderness. Nonetheless, patients with GAS puerperal sepsis may have an unusual presentation, where fever is absent, signs and symptoms can be mild, non-specific, and not indicative of the severity of infection. This unusual presentation may lead to a delayed diagnosis and increase the risk of severe puerperal sepsis. Therefore, in these cases, a high index of suspicion, early escalation of care, and prompt early antibiotic and surgical treatment are crucial to saving the parturient’s life (15–21).

## Data availability statement

The original contributions presented in this study are included in the article/supplementary material, further inquiries can be directed to the corresponding author.

## Author contributions

DK, RA, GG, SP, and YE conceived and designed the case report. DP helped with data acquisition. DK wrote the draft manuscript. All authors read the draft manuscript and made significant intellectual contributions to the final version and were responsible for the integrity of the data and accuracy of the analysis, and approved the final version of the manuscript for submission.
